# 30‐Day DAPT in Patients at High Bleeding Risk Undergoing PCI With Biodegradable‐Polymer Sirolimus‐Eluting Ultra‐Thin Stent

**DOI:** 10.1002/ccd.31481

**Published:** 2025-03-10

**Authors:** Andrea Erriquez, David M. Leistner, Valeria Paradies, Rita Pavasini, Matteo Serenelli, Gianni Casella, Simone Biscaglia, Christoph Naber, Gianluca Campo, Pieter C. Smits

**Affiliations:** ^1^ Cardiology Unit, Azienda Ospedaliero Universitaria di Ferrara Cona Ferrara Italy; ^2^ Department of Cardiology, Angiology and Intensive Care Medicine Goethe University Hospital Frankfurt Germany; ^3^ Department of Cardiology Maasstad Hospital Rotterdam the Netherlands; ^4^ Cardiology Unit, Ospedale Maggiore Bologna Italy; ^5^ Facharztpraxis Baldeney Essen Germany

**Keywords:** dual antiplatelet therapy, high beeding risk, meta‐analysis, percutaneous coronary intervention, sirolimus‐eluting biodegradable polymer stent

## Abstract

**Background:**

There is limited evidence on the safety and efficacy of biodegradable‐polymer sirolimus‐eluting ultra‐thin stent (BP‐SES) in patients at high bleeding risk (HBR) undergoing percutaneous coronary intervention (PCI).

**Aims:**

This study aims to evaluate the clinical outcomes of HBR patients treated with BP‐SES and ≤ 30‐day dual antiplatelet therapy (DAPT) regimen.

**Methods:**

A systematic review was conducted to identify relevant studies involving HBR patients who underwent PCI with BP‐SES (Supraflex Cruz). Individual patient‐level data were extracted from the included studies. The primary endpoint was the composite of cardiovascular death, myocardial infarction, or clinically driven target lesion revascularization at 1‐year. The safety endpoint was the 1‐year occurrence of Bleeding Academic Research Consortium (BARC) type 3−5.

**Results:**

The study population included 1691 patients. Of these, 928 patients (55%) received a ≤ 30‐day DAPT, while 763 patients (45%) received a longer DAPT regimen. In the ≤ 30‐day DAPT group, primary outcome events occurred in 89 patients (9.5%, 95% CI: 7.7%−11.6%). The upper limit of the one‐sided 95% CI of 11.6% was below the pre‐specified non‐inferiority margin of 14%. There was no significant difference in the primary endpoint between the ≤ 30‐day DAPT group and the >30‐day DAPT group (propensity score adjusted HR: 0.95, 95% CI: 0.67−3). Notably, the incidence of BARC 3−5 bleeding events was significantly lower in the ≤ 30‐day DAPT group.

**Conclusions:**

In HBR patients treated with BP‐SES, a ≤ 30‐day DAPT regimen is associated with a low rate of ischemic events and a significant reduction in major bleeding events.

**Trial Registration:** PROSPERO CRD42024524208.

## 1 Introduction

Advancements in percutaneous coronary intervention (PCI) have made it possible to treat older, sicker, and more comorbid patients. As a result, it is unsurprising that over one‐third of patients undergoing PCI are at high bleeding risk (HBR) [1, 2]. Numerous studies have demonstrated that, in HBR patients, the protective benefit of dual antiplatelet therapy (DAPT) is often outweighed by bleeding complications, undermining the overall benefit‐risk balance [1, 2]. Consequently, consensus guidelines recommend short (< 3‐month, possibly ≤ 1‐month) DAPT regimen in this population [3]. The latest generations of drug‐eluting stents (DES) have shown an improved safety profile, even as DAPT durations have been progressively reduced from 12 months to ≤ 30 days. However, this evidence primarily stems from studies including stents with strut thicknesses ≥ 80 μm [4, 5]. Few studies investigated the safety of ≤ 30‐day DAPT regimen with ultra‐thin (≤ 60 μm) DES [6, 7], which might show additional benefit compared to thicker strut stents.

The Supraflex Cruz (Sahajanand Medical Technologies Ltd.) is a biodegradable‐polymer sirolimus‐eluting ultra‐thin stent (BP‐SES). Previous studies have demonstrated its non‐inferiority to the gold‐standard durable‐polymer everolimus‐eluting stent (DP‐EES) [8], with favorable outcomes in terms of stent thrombosis and in‐stent restenosis, even in patients with complex coronary artery disease [9, 10, 11, 12]. However, robust evidence about its efficacy and safety with ≤ 30‐day DAPT remains limited.

Given the clinical importance of understating and demonstrating the safety and efficacy of (BP‐SES) in HBR patients undergoing PCI and receiving ≤ 30‐day DAPT, we conducted an individual patient meta‐analysis of clinical studies including HBR patients treated with Supraflex Cruz.

## 2 Methods

**Figure 1:**
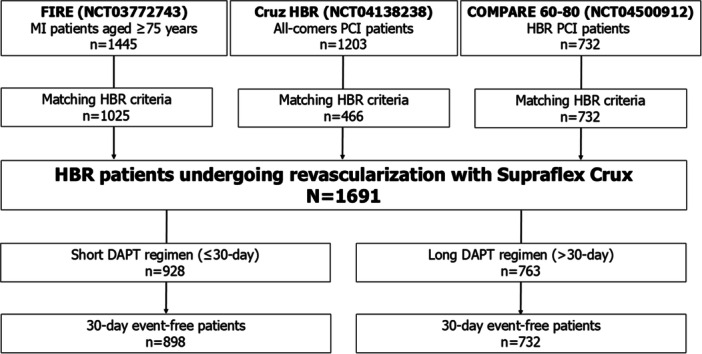
Study flowchart. COMPARE 60−80, Comparison of the Supraflex Cruz 60‐micron stent strut versus the Ultimaster Tansei 80‐micron stent strut in HBR PCI population. DAPT, dual antiplatelet therapy; HBR, high bleeding risk; FIRE, Functional Assessment in Elderly MI Patients with Multivessel Disease.

The Evidence Accumulated Regarding Therapeutic strategy in HBR patients (EARTH‐HBR) individual patient meta‐analysis was performed in accordance with the Preferred Reporting Items for Systematic Reviews and Meta‐Analyses (PRISMA) guidelines. The protocol was registered in the PROSPERO registry before the start of the analyses (id: CRD42024524208). A comprehensive search was performed using PubMed, Google Scholar, Biomed Central, and Cochrane databases to identify clinical studies involving HBR patients who underwent PCI with implantation of the BP‐SES Supraflex Cruz (Supporting Information S1: Figure 1). The search terms included: (HBR AND PCI AND [Supraflex Cruz stent]) (the full search strategy is available in the Supporting Information S1: Material online). Clinical studies enrolling fewer than 20 patients, as well as abstracts, conference presentations, or studies not published in English or in peer‐reviewed journals, were excluded from the analysis. Two independent reviewers (G.C. and R.P.) screened the studies based on the search criteria. Any disagreements regarding study inclusion were resolved through discussion with a third reviewer (S.B.). All studies were carefully examined to eliminate any irrelevant duplicates. Ethics committees at each participating site approved the study protocols, and all patients provided written informed consent. The primary objective of this individual patient meta‐analysis was to demonstrate that the 1‐year incidence of ischemic outcomes in patients treated with BP‐SES and ≤ 30‐day DAPT regimen is non‐inferior to a pre‐established cutoff based on previous studies [1, 2, 3, 4, 5, 6]. The secondary objective was to compare the rate of ischemic and bleeding outcomes in patients treated with BP‐SES and receiving ≤ 30‐day DAPT versus those receiving a longer DAPT regimen.

### 2.1 Study Population

The corresponding authors of the clinical studies that met the search criteria were contacted to obtain individual patient‐level data. From the original database of each clinical study (Supporting Information S1: Tables 1–2), patients were selected based on the following criteria: (i) HBR, and (ii) PCI and implantation of BP‐SES Supraflex Cruz. The criteria for defining HBR were established in accordance with the Academic Research Consortium HBR (ARC‐HBR) document, and both major and minor HBR criteria were systematically collected within the electronic Case Report Form (eCRF) by the investigators [3]. Patients were categorized as HBR if they fulfilled at least one major criterion or two minor criteria [3]. On top of this, for each patient, the PRECISE DAPT and the ARC‐HBR scores were calculated (for the ARC‐HBR score 1 point was attributed for each major criterion and 0.5 point for each minor criterion). Duration of the DAPT regimen was at the discretion of the investigators. However, each study protocol recommended, in agreement with available consensus document [3], a ≤ 30‐day DAPT for HBR patients. In cases where oral anticoagulant therapy was indicated, the study protocols recommended dual antithrombotic therapy, consisting of clopidogrel and a novel oral anticoagulant. A common data set was created, including baseline patient characteristics, angiographic data, treatment details, and clinical outcomes. These data were reviewed by each trial investigator and cross‐referenced with previously published results from the respective studies.

### 2.2 Study Endpoints

The primary outcome was the device‐oriented composite endpoint (DOCE), defined as the composite of cardiovascular death, any myocardial infarction (not clearly attributable to a nontarget vessel), or clinically‐driven target lesion revascularization. Secondary outcomes included the individual components of the primary endpoint, cardiovascular death or MI, all‐cause mortality, any stroke, any MI, any revascularization, and definite and probable stent thrombosis. Safety endpoints focused on Bleeding Academic Research Consortium (BARC) type 3−5 bleedings. Endpoint definitions used in each trial adhered to those used in the original trials and are provided in Supplementary material (Supporting Information S1: Table 3). In each study, adverse events were adjudicated by an independent blinded committee. These independent blind committees also determined whether MI and revascularization events were attributable to the target vessel, specifically the vessel treated with the Supraflex Cruz stent.

### 2.3 Data Analysis

G.C. and M.S. had full access to all the data and took responsibility for its integrity and data analysis. The data underlying this article can be requested from the EARTH‐HBR study Executive Committee under reasonable conditions. For the primary analysis, which examined the occurrence of the DOCE in HBR patients treated with ≤ 30‐day DAPT, it was assumed that the 1‐year DOCE occurrence would be approximately 10%, based on the risk profile of the population [1, 2, 3, 4, 5, 6]. A non‐inferiority margin of 4% was established, in line with previous similar studies. To achieve 80% power for demonstrating non‐inferiority with a one‐sided type I error of 0.05, a minimum study population of 696 HBR patients treated with the BP‐SES Supraflex Cruz and receiving ≤ 30‐day DAPT was required. The analysis used a one‐stage approach, which simultaneously included all data from the trials. Continuous variables were summarized with means (SD) or median [IQR], and comparisons were made using the Student *t*‐test or the Wilcoxon test, as appropriate. Categorical variables were presented as frequencies and percentages, and comparative analyses were conducted using either the Pearson chi‐square or Fisher's exact test, as appropriate. Time‐to‐event data were assessed using Kaplan–Meier estimates and Cox proportional‐hazards models. The proportionality assumption was tested using Schoenfeld residuals and was confirmed (*p* > 0.05 for all outcomes). Due to the observed disparities in characteristics between ≤ 30‐day DAPT and > 30‐day DAPT groups, a propensity score (PS) model was developed. This model used a logistic regression model, incorporating significant variables identified during the analysis. The score was then included as an adjustment covariate in the multilevel Cox proportional hazards model to assess the association between ≤ 30‐day DAPT versus > 30‐day DAPT and outcomes. Statistical analyses were performed using the STATA statistical software version 16 (StataCorp LLC, College Station, TX, USA).

## 3 Results

**Figure 2:**
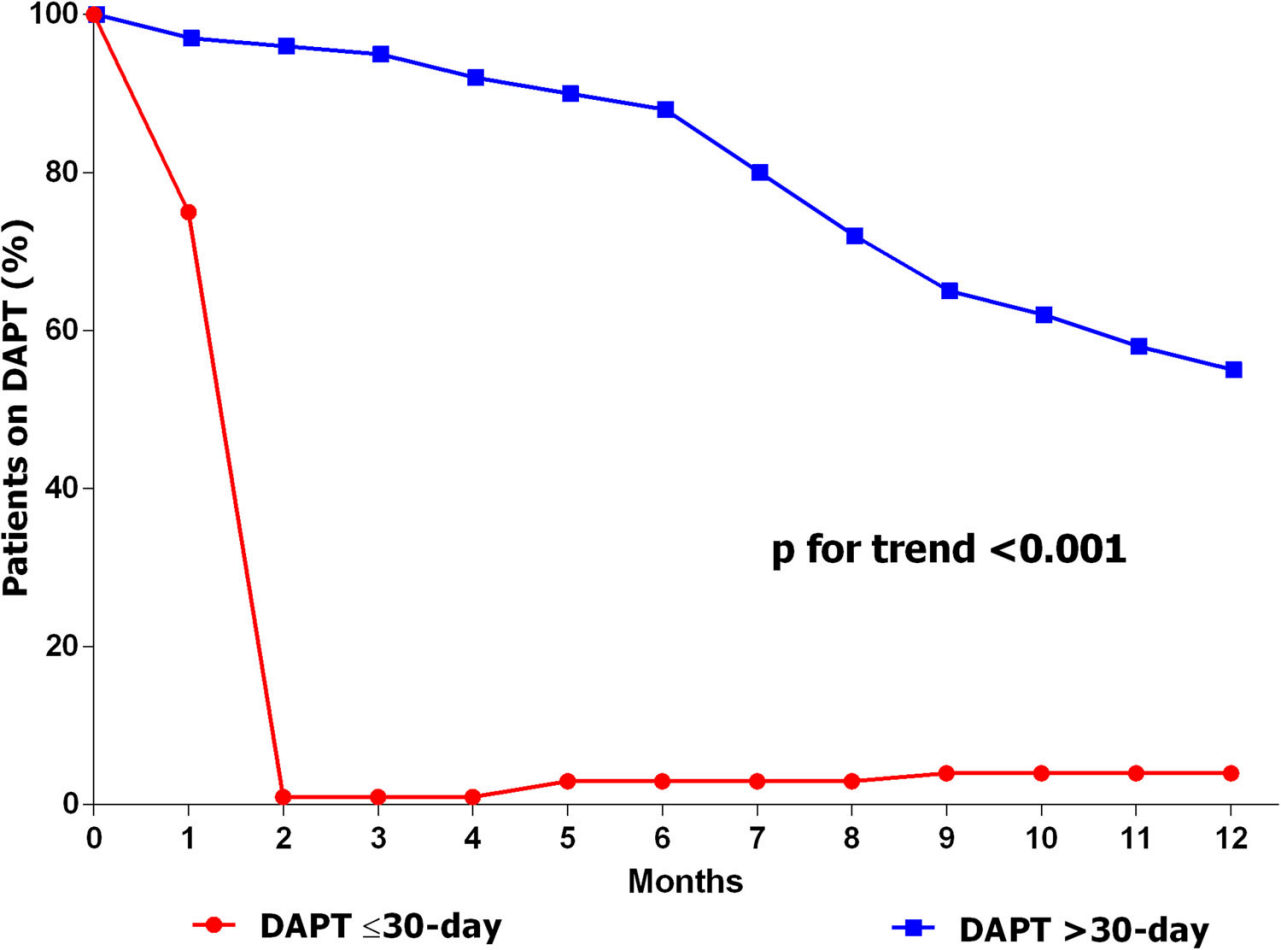
Rate of adherence to DAPT in patients. Blue: >30‐day DAPT group. Red: ≤30 DAPT group. DAPT, dual antiplatelet therapy. [Color figure can be viewed at wileyonlinelibrary.com]

The search strategy was carried out in April 2024, yielding 76 results (Supporting Information S1: Figure 1). Three studies met the selection criteria: Cruz HBR [13], Functional Assessment in Elderly MI Patients with Multivessel Disease (FIRE) [10], and Comparison of the Supraflex Cruz 60‐micron stent strut versus the Ultimaster Tansei 80‐micron stent strut in HBR PCI population (COMPARE 60/80 HBR) [7]. As shown in Figure 1, the three studies enrolled 3380 patients of whom 2223 (66%) met the ARC‐HBR definition (Figure 1). Among these, 1691 (76%) were treated exclusively with BP‐SES for all coronary lesions requiring PCI (Figure 1). The median age of the population was 79 years [75−83], with ages ranging from 40 to 90 years, and 599 patients (35%) were female. The median ARC‐HBR average score was 2 [1, 2], with values ranging from 1 to 5. The median PRECISE‐DAPT value was 30 [24−36], with values ranging from 13 to 83. The majority of patients (*n* = 1015, 60%) were hospitalized for MI. Overall, 928 patients (55%) received a DAPT regimen of ≤ 30 days, while 763 patients (45%) received a longer DAPT regimen (Table 1). DAPT prescription patterns over time in the two study groups are depicted in Figure 2 (red line, ≤ 30‐day DAPT; blue line > 30‐day DAPT). Baseline, procedural, and treatment characteristics significantly differ between the two groups (Table 1).

**Table 1:** Baseline characteristics of HBR populations.

Characteristic	All (*n* = 1691)	≤ 30‐day DAPT (*n* = 928)	> 30‐day DAPT (*n* = 763)	*p*
Age—ears	80 [75−84]	80 [76−84]	79 [75−83]	< 0.001
Male sex—no. (%)	1092 (64)	587 (63)	505 (66)	0.228
Medical history—no. (%)				
Hypertension	1412 (83)	759 (82)	653 (86)	0.042
Dyslipidemia	981 (58)	514 (55)	467 (61)	0.018
Diabetes	531 (31)	268 (29)	263 (35)	0.015
Current smoker	141 (8)	71 (8)	70 (9)	0.298
Prior MI	500 (29)	282 (30)	218 (29)	0.446
Prior PCI	630 (37)	308 (33)	322 (42)	< 0.001
History of AF	317 (19)	241 (26)	76 (10)	< 0.001
eGFR < 60 mLmin	958 (57)	527 (57)	431 (56)	0.94
PAD	316 (19)	168 (18)	148 (19)	0.537
CVA	174 (10)	99 (11)	75 (10)	0.628
Clinical presentation				
STEMI—no. (%)	283 (16.7)	206 (22)	77 (10)	< 0.001
NSTEMI—no. (%)	732 (43)	461 (50)	271 (35)	
CCS—no. (%)	676 (40)	261 (28)	415 (55)	
LVEF—%	50 [40‐58]	50 [40‐58]	50 [40‐58]	0.460
Radial access—no. (%)	1481 (87)	865 (93)	616 (81)	< 0.001
Treated vessels	(*n* = 2583)	(*n* = 1577)	(*n* = 1006)	
Median [IQR]	2 [1−2]	2 [1−2]	2 [1−2]	0.567
Left main coronary artery	84 (3)	48 (3)	36 (4)	< 0.001
Left anterior descending artery	890 (34)	561 (35)	329 (33)	
Circumflex artery	769 (30)	431 (27)	338 (33)	
Right coronary artery	782 (30)	501 (33)	281 (28)	
Ramus Intermedius artery	58 (22)	36 (2)	22 (2)	
Antithrombotic drugs at discharge—no. (%)	(*n* = 1679)	(*n* = 926)	(*n* = 753)	
Aspirin	1467 (87)	716 (77)	751 (98)	< 0.001
Clopidogrel	1447 (86)	810 (87)	637 (82)	0.103
Newer P2Y12i	232 (14)	116 (13)	116 (15)	
Vitamin K antagonist	121 (7)	63 (7)	58 (7)	0.654
Novel oral anticoagulant	591 (35)	402 (43)	189 (24)	< 0.001
Triple antithrombotic therapy	505 (30)	258 (28)	247 (33)	< 0.001
Dual antithrombotic therapy	207 (12)	207 (22)	0 (0)	
Dual antiplatelet therapy	965 (57)	461 (50)	504 (66)	

Abbreviations: AF, atrial fibrillation; CCS, chronic coronary syndrome; CVA, cerebrovascular accident; LVEF, left ventricular ejection fraction; MI, myocardial infarction; NSTEMI, no ST‐segment elevation MI; PAD, peripheral artery disease; PCI, percutaneous coronary intervention; STEMI, ST‐segment elevation MI.

### 3.1 Ischemic Outcomes in ≤ 30‐day DAPT Group

**Figure 3:**
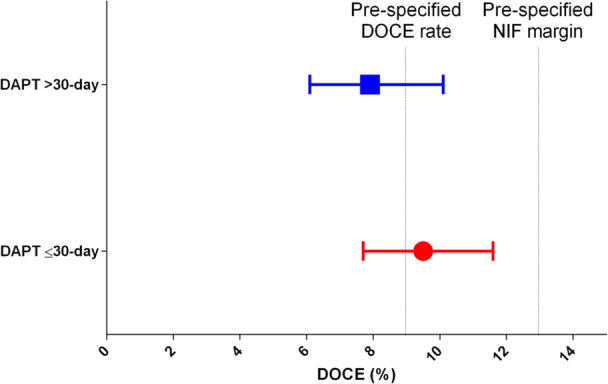
Occurrence of DOCE in the study groups. The dot and horizontal lines show the occurrence of DOCE (95%CI) in patients of the study groups. The vertical lines represent the pre‐specified DOCE rate and non‐inferiority (NIF) margin. Blue: >30‐day DAPT group. Red: ≤ 30 DAPT group. [Color figure can be viewed at wileyonlinelibrary.com]

In HBR patients treated with ≤ 30‐day DAPT, 50 (5.3%) cardiovascular death, 33 (3.5%) MI and 43 (4.6) clinically‐driven target lesion revascularization occurred at 1‐year (Table 2). A primary outcome event occurred in 89 patients (9.5%, 95% CI 7.7%−11.6%). The upper limit of the one‐sided 95% CI of 11.6% was below the pre‐specified 14% required demonstrating the non‐inferiority (p for non‐inferiority < 0.001) (Figure 3). The occurrence of the secondary ischemic endpoints was reported in Table 2. Definite/probable stent thrombosis was observed in 12 patients (1.2%, 0.6%−2.2%) (Table 2). Notably, only 4 (0.4%, 95% CI 0.1%−1.1%) definite stent thrombosis occurred after the 30‐day window.

**Table 2:** Clinical outcomes.

Outcome	≤ 30‐day DAPT (*n* = 928) no. (%)	> 30‐day DAPT (*n* = 763) no. (%)	Unadjusted HR (95% CI)	Propensity‐matched adjHR (95% CI)
*Primary outcome*				
Composite of CV death, myocardial infarction, or clinically‐driven TLR	89 (9.5)	60 (7.8)	1.21 (0.87−1.68)	0.95 (0.67−1.33)
*Secondary outcomes*				
CV death or MI	77 (8.3)	45 (5.9)	1.40 (0.97−2.02)	1.10 (0.75−1.62)
Death	97 (10.4)	79 (10.3)	1.01 (0.74−1.34)	0.86 (0.63−1.18)
Cardiovascular death	50 (5.3)	32 (4.1)	1.27 (0.81−1.98)	0.92 (0.58−1.46)
Myocardial infarction	33 (3.5)	17 (2.2)	1.06 (0.69−1.62)	0.99 (0.63−1.56)
Clinically‐driven TLR	43 (4.6)	29 (3.8)	1.23 (0.77−1.97)	1.05 (0.64−1.73)
Any revascularization	49 (5.2)	44 (5.7)	0.92 (0.61−1.39)	0.85 (0.55−1.31)
Stroke	11 (1.2)	17 (2.2)	0.53 (0.25−1.13)	0.45 (0.20−1.13)
Definite/probable stent thrombosis	12 (1.2)	10 (1.2)	1.01 (0.51−2.23)	1.17 (0.48−2.88)
Definite stent thrombosis	10 (1)	8 (1)	1.03 (0.40−2.59)	1.33 (0.49−3.61)
Probable stent thrombosis	2 (0.2)	2 (0.2)	0.82 (0.11−5.77)	0.67 (0.08−5.32)
*Safety outcomes*				
BARC 3−5	28 (3)	57 (7.5)	0.39 (0.25−0.62)	0.34 (0.21−0.54)

Abbreviations: BARC, Bleeding academic Research Consortium; CV, cardiovascular; DAPT, dual antiplatelet therapy; HR, hazard risk; MI, myocardial infarction; TLR, target lesion revascularization; 95%CI, 95% confidence interval.

### 3.2 Ischemic Outcomes According to DAPT Groups

**Figure 4:**
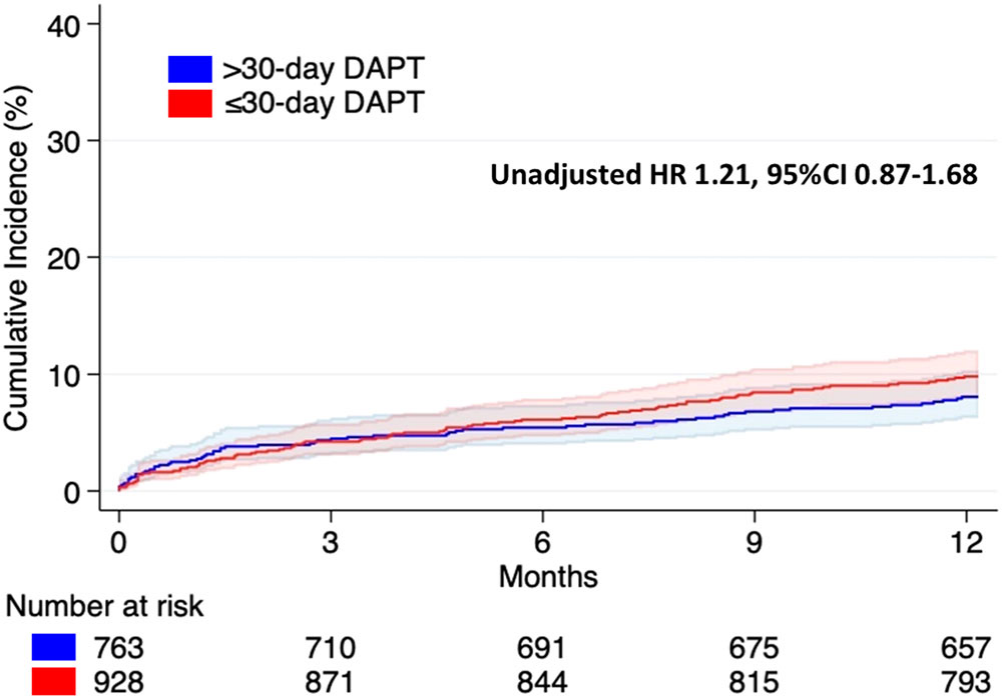
Cumulative occurrence of DOCE stratified according to DAPT length. Solid lines: cumulative occurrence in the study groups. Colored areas: 95% confidence interval in the study subgroups. Blue: > 30‐day DAPT group. Red: ≤ 30‐day DAPT group. [Color figure can be viewed at wileyonlinelibrary.com]

The occurrence of ischemic events in HBR patients treated with > 0‐day a DAPT regimen is reported in Table 2 and Figure 3. The occurrence of the primary endpoint did not differ between the two groups (9.5% in the ≤ 30‐day DAPT vs. 7.8% in the > 30‐day group, unadjusted HR 1.21, 95% CI 0.87−1.68) (Table 2 and Figure 4). No significant difference was observed in any of the secondary ischemic endpoints between the groups (Table 2), including the occurrence of definite/probable stent thrombosis (1.2% in the ≤ 30‐day DAPT vs. 1.2% in the > 30‐day group, unadjusted HR 1.01, 95% CI 0.51−1.23). After adjusting the analyses using a PS model ‐ which included variables such as age, arterial hypertension, dyslipidemia, prior PCI, history of atrial fibrillation, clinical presentation, and treated vessels—no significant differences were observed between the ≤ 30‐day DAPT and > 30‐day DAPT groups (adjusted HR 0.95, 95% CI 0.67−1.33) (Table 2). This finding was consistent across the other ischemic outcomes as well (Table 2).

### 3.3 Bleeding Outcomes According to DAPT Groups

**Figure 5:**
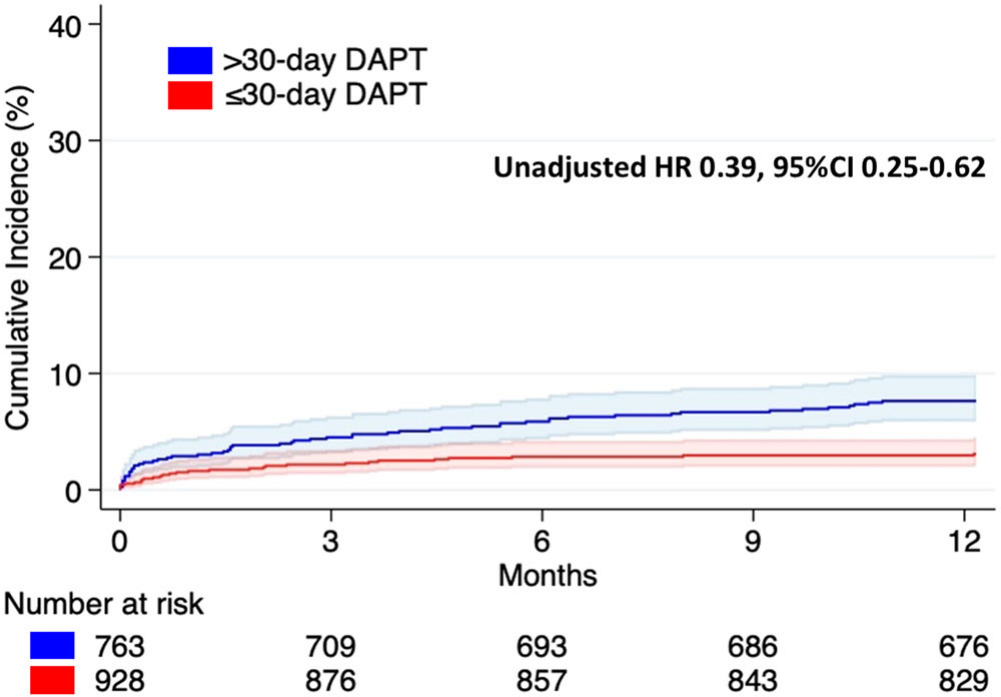
Cumulative occurrence of BARC 3−5 bleedings stratified according to DAPT length. Solid lines: cumulative occurrence in the study groups. Colored areas: 95% confidence interval in the study subgroups. Blue: > 30‐day DAPT group. Red: ≤ 30 DAPT group. [Color figure can be viewed at wileyonlinelibrary.com]

A total of 28 patients in the ≤ 30‐day DAPT group and 57 patients in the > 30‐day DAPT group experienced a BARC 3−5 bleeding event (Table 2). The incidence of BARC 3−5 bleeding was significantly lower in the ≤ 30‐day DAPT group (unadjusted HR 0.39, 95% CI 0.25−0.62) (Table 2 and Figure 5). This finding remained consistent after adjustment using the PS model (adjusted HR 0.34, 95% CI 0.21−0.54) (Table 2).

## 4 Discussion

This meta‐analysis, based on individual patient‐level data from three studies including 1691 HBR patients treated with implantation of BP‐SES [7, 10, 13], yielded several key findings. First, patients treated with BP‐SES and ≤ 30‐day DAPT showed a low 1‐year occurrence of DOCE, non‐inferior to prespecified cut‐off based on similar studies with different stent technology. Second, in a direct comparison of BP‐SES patients receiving ≤ 30‐day DAPT versus those on longer DAPT regimen, there was no significant difference in the rate of ischemic endpoints, including stent thrombosis. Third, a ≤ 30‐day DAPT regimen, when compared to longer ones, was associated with a significant reduction in bleeding outcomes.

In recent years, several studies have highlighted the negative prognostic implication of bleeding adverse events following PCI [14]. Specifically, a BARC 3−5 adverse event has been shown to carry the same prognostic weight for all‐cause mortality as a recurrent MI [14]. For this reason, considerable efforts have been directed toward identifying patients who are more likely to experience bleeding complications during DAPT, as opposed to ischemic events, and determining the optimal DAPT duration that balances the risks of bleeding and ischemia.

The development of the ARC‐HBR classification has standardized the identification of HBR patients, providing clinicians with a practical tool to assess bleeding risk [3]. Additionally, the Management of HBR Patients Post Bioresorbable Polymer Coated Stent Implantation with an Abbreviated versus Standard DAPT Regimen (MASTER DAPT) trial confirmed that a short DAPT regimen (30−44 days, median 34 days) is optimal for HBR patients, offering significant reductions in bleeding events without a concomitant increase in ischemic complications [15].

The initial studies evaluating the benefit of short DAPT regimen were conducted in patients treated with polymer‐free DES, based on the hypothesis that the stent polymer would carry a pro‐thrombogenic effect [4]. However, subsequent studies have shown that both durable‐ and resorbable‐polymer DES are effective and safe with short DAPT [5, 6, 7]. The Supraflex Cruz is an ultra‐thin (60 µm in all the stent diameters) cobalt‐chromium stent coated with biodegradable polymer and realizing sirolimus. The average coating thickness of Supraflex Cruz is between 4 μm and 6 μm. Previous studies have shown that Supraflex Cruz is non‐inferior to durable‐polymer DES in an all‐comer PCI population [8]. Specifically concerning HBR patients, it has demonstrated non‐inferiority in a head‐to‐head comparison with a thin‐strut biodegradable‐polymer sirolimus‐eluting stent, which was also utilized in the MASTER DAPT trial [7]. However, it is important to note that, in this trial, not all patients were treated with ≤ 30‐day DAPT and it was not powered to confirm the efficacy and safety of the stent with this DAPT regimen [7]. Consequently, robust evidence demonstrating the efficacy and safety of Supraflex Cruz with ≤ 30‐day DAPT remains absent.

This individual‐patient meta‐analysis has been conducted to fill this gap. From three different studies, individual data of HBR patients treated with BP‐SES was collected. The criteria for ARC‐HBR were reviewed and confirmed by an independent group of physicians. Adverse events, which were adjudicated in each trial, were reviewed and screened for their association with the stent. Finally, the population was divided into HBR patients treated with ≤ 30‐day DAPT and those receiving longer DAPT, enabling comparisons between groups concerning ischemic and bleeding outcomes. The findings are consistent: patients treated with ≤ 30‐day DAPT exhibited a low occurrence of ischemic events, with no signals of concern regarding stent thrombosis, while also significantly reducing BARC 3−5 bleeding complications.

To better contextualize these findings, it is important to compare our patients with those in previous similar studies. Our cohort is older, with a median age of 80 years compared to a mean age of 75 years in prior studies and has a higher percentage of female participants (37% vs. around 30%) [4, 5, 6]. The bleeding risk profile of the population is high, as supported by the value of ARC‐HBR score (median 2 [1, 2], mean 1.8 ± 0.7, vs. 1.6 ± 0.7 in the COMPARE 60−80) or by the PRECISE‐DAPT score (median 30 [15−83], mean 31 ± 11, vs. 27 ± 11 in the MASTER‐DAPT). Additionally, 22% of our population consisted of STEMI patients, who are considered at higher ischemic risk, a figure that is substantially higher than in many previous studies, where STEMI patients were either excluded [5], or comprised only 1.7%−6% of the population [4, 6]. In general, the number of patients admitted for acute coronary syndromes is higher, being around 70%, as compared to 17%−45% of the previous ones [4, 5, 6]. Moreover, the mean number of treated vessels per patient in our study was greater (1.7 vs. 1.3 in previous studies) [4, 5, 6]. Despite these high‐risk characteristics, the performance of the Supraflex Cruz stent was commendable, with a relatively low rate of ischemic complications; specifically, there were only 12 cases of stent thrombosis.

Our data carry two significant implications. First, the EARTH‐HBR meta‐analysis underscores the importance of accurately identifying HBR patients and treating them with a ≤ 30‐day DAPT regimen. This very short regimen may guarantee the best balance between ischemic and bleeding outcomes. Second, the MATRIX‐2 trial (Monotherapy with P2Y12 Inhibitors in Patients With Atrial fIbrillation Undergoing Supraflex Stent Implantation, NCT05955365) has commenced recruitment, aiming to evaluate the safety and efficacy of a monotherapy regimen with a P2Y12 inhibitor for 1 month, followed by direct oral anticoagulant (DOAC) monotherapy, compared to current standard care in atrial fibrillation patients undergoing PCI. The strong performance of the Supraflex Cruz stent, coupled with the very low rate of stent thrombosis observed in this meta‐analysis, further supports the rationale for the MATRIX‐2 trial.

### 4.1 Study Limitations

This study has certain limitations that must be acknowledged. First, the DAPT regimen was not randomized, as the final decision rested with the investigator. Second, only the COMPARE 60−80 trial randomized patients to two different stent technologies [7], which means that any indirect comparisons with other stents should be regarded as hypothesis‐generating. Third, the overall rate of DOCE may appear high, particularly in comparison to similar studies; however, considering the mean age, risk profile, and percentage of MI patients, this is in line with expectations. Finally, while our database included the most relevant variables, we cannot exclude the potential presence of confounding factors, such as ethnicity or race, that may not have been captured in our analysis.

## 5 Conclusions

In HBR patients undergoing PCI with a biodegradable‐polymer ultra‐thin sirolimus‐eluting stent, a short DAPT regimen (≤ 30 days) appears to be effective and safe, yielding a low incidence of ischemic outcomes while minimizing bleeding occurrences.

## Conflicts of Interest

The authors declare no conflicts of interest.

## Supporting information

Supporting information.

## Data Availability

The data that support the findings of this study are available on request from the corresponding author. The authors have nothing to report.
