# Supplementary material for: 30‐Day DAPT in Patients at High Bleeding Risk Undergoing PCI With Biodegradable‐Polymer Sirolimus‐Eluting Ultra‐Thin Stent
Source: Catheter Cardiovasc Interv. 2025 Mar 10;105(6):1502–9. doi: 10.1002/ccd.31481 (PMC12057321; doi:10.1002/ccd.31481)
Supplement: Supplementary file 1 — Supporting information. [file CCD-105-1502-s001.docx]

**SUPPLEMENTAL MATERIAL**

- **Search strategy details page 2**
- **Supplemental figure 1 page 3**
- **Supplemental table 1 page 4**
- **Supplemental table 2 page 5**
- **Supplemental table 3 page 7**
- **Quality appraisal page 9**
- **Supplemental figure 2 page 10**
- **Supplemental table 4 page 11**
- **Publication bias analysis page 12**
- **Supplemental table 5 page 12**
- **Supplemental figure 3 page 12**
- **Supplemental Table 6. Sensitivity analysis page 13**

**Full search strategy details**

"high"[All Fields] AND ("bleedings"[All Fields] OR "hemorrhage"[MeSH Terms] OR "hemorrhage"[All Fields] OR "bleed"[All Fields] OR "bleeding"[All Fields] OR "bleeds"[All Fields]) AND ("risk"[MeSH Terms] OR "risk"[All Fields]) AND ("high"[All Fields] AND ("bleedings"[All Fields] OR "hemorrhage"[MeSH Terms] OR "hemorrhage"[All Fields] OR "bleed"[All Fields] OR "bleeding"[All Fields] OR "bleeds"[All Fields]) AND ("risk"[MeSH Terms] OR "risk"[All Fields]) AND ("percutaneous coronary intervention"[MeSH Terms] OR ("percutaneous"[All Fields] AND "coronary"[All Fields] AND "intervention"[All Fields]) OR "percutaneous coronary intervention"[All Fields]) AND ("Supraflex"[All Fields] AND "Cruz"[All Fields] AND ("stent s"[All Fields] OR "stentings"[All Fields] OR "stents"[MeSH Terms] OR "stents"[All Fields] OR "stent"[All Fields] OR "stented"[All Fields] OR "stenting"[All Fields]))) AND ("Supraflex"[All Fields] AND "Cruz"[All Fields] AND ("stent s"[All Fields] OR "stentings"[All Fields] OR "stents"[MeSH Terms] OR "stents"[All Fields] OR "stent"[All Fields] OR "stented"[All Fields] OR "stenting"[All Fields]))

**Supplemental Figure 1. PRISMA flow diagram**

**
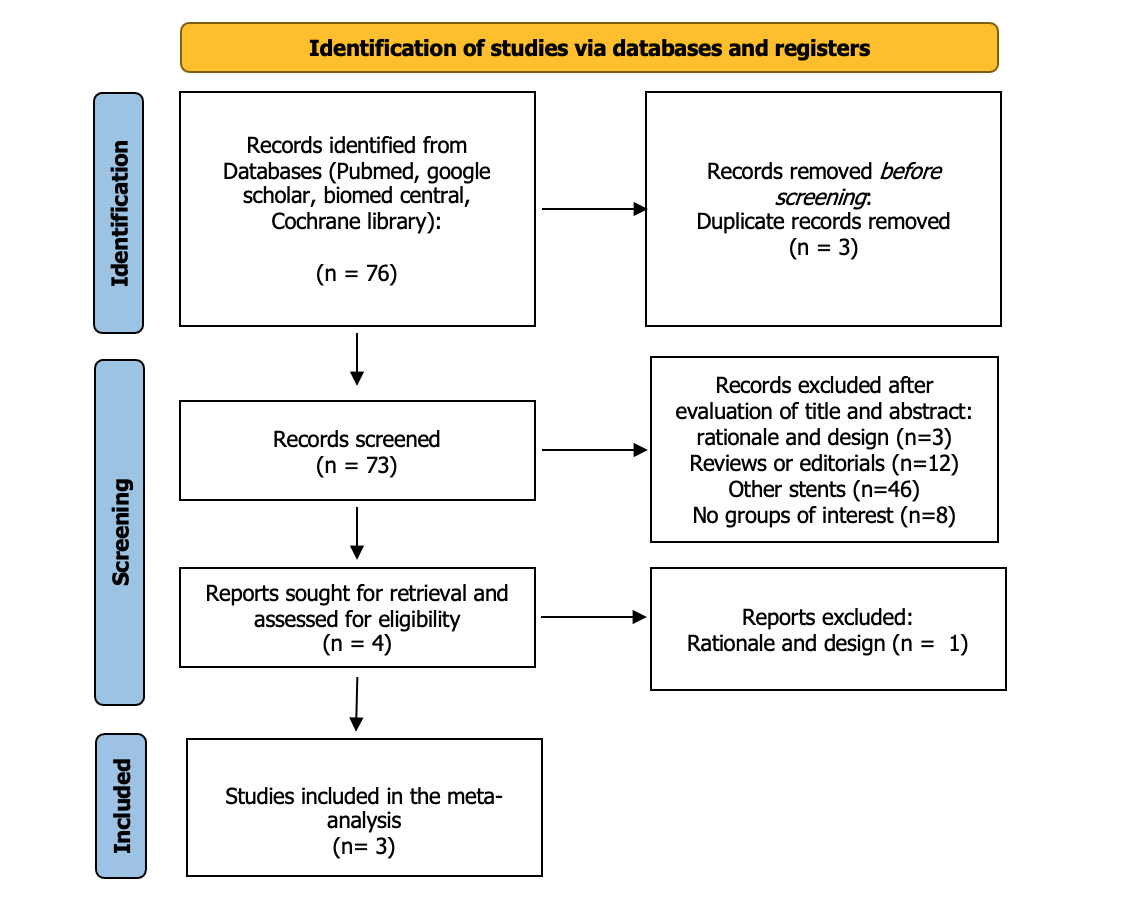
**

**Supplemental Table 1. Study characteristics**

| **References** | **Blinded adjudication committee** | **Trial registration** | **Entire study population** | **Pts meeting criteria for the present analysis** | **Median and longest FU** | **Multicentric; multinational** | **Time of the enrollment** | **Geographic area** |
| --- | --- | --- | --- | --- | --- | --- | --- | --- |
| ***Cruz HBR*** | Y | NCT04138238 | 1203 | 460 | 1 year | Y; Y | 26 February 2020 - 22 October 2020 | Switzerland, Germany and France |
| ***FIRE*** | Y | [NCT03772743](http://clinicaltrials.gov/show/NCT03772743) | 1445 | 865 | 1 year | Y; Y | 18 July 2019 - 25 October 2021 | Italy, Spain, Poland |
| ***COMPARE 60/80 HBR*** | Y | NCT04500912 | 741 | 366 | 1 year | Y; N | September 2020 - August 2022 | The Netherlands |

Y: yes; N: no.

**Supplemental Table 2. Inclusion and exclusion criteria and primary and secondary outcomes of included studies.**

| **References** | **Inclusion Criteria** | **Exclusion criteria** | **Primary outcome** | **Secondary outcome** |
| --- | --- | --- | --- | --- |
| ***Cruz HBR*** | Patients ≥ 18 years old; De novo or restenotic significant stenosis in at least one native coronary artery; Patients with silent ischemia, stable angina, unstable angina or non STEMI eligible for PCI (no limitation of the number of treated lesions and vessels, except higher tercile of Syntax score assessed by the site); Target lesions suitable for PCI with Drug Eluting Stent diameter between 2.00 and 4.50 mm; Total lesion length should be from 15 to 120 mm; Patient is willing and capable to sign the written informed consent and comply with all requirements of the registry; Planned staged procedures are allowed within 3 months using Supraflex CruzTM stent only. | SYNTAX Score > 32; Hemodynamic instability or cardiogenic shock; Known hypersensitivity or contraindication to any component of the study stent or the eluting drug, to media contrast, to dual antiplatelet therapy medication required by current practice; Subject is pregnant, nursing or is a woman with childbearing potential; Any co morbid condition with life expectancy < 1 year or that may result in protocol noncompliance; Patients who are participating in another drug or device investigational study, which has not reached its primary endpoint; Patients under judicial protection, tutorship or curatorship | Device oriented composite endpoint, a composite of cardiovascular death, MI not clearly attributable to a non-target vessel and clinically driven target lesion revascularization at 12 months | Rates of any MI, any death (both cardiovascular and non-cardiovascular), any revascularization, stent thrombosis (ST) and major bleeding BARC (BARC 3, 4 or 5 for HBR patients) at six and 12 months |
| ***FIRE*** | STEMI or NSTEMI aged 75 years and older, successful PCI of the culprit lesion, and had multivessel disease with at least one lesion in a non-culprit coronary artery that had a minimum vessel diameter of 2.5 mm and a visually estimated diameter stenosis of 50 to 99%. | Inability to identify a clear culprit lesion, localization of the non-culprit lesion in the left main coronary artery, planned or previous surgical revascularization, or life expectancy of less than one year. | composite of death, myocardial infarction, stroke, or any revascularization at one year. | Composite of cardiovascular death or myocardial infarction, single component of the primary and secondary outcomes, non-cardiovascular death, cerebrovascular accident, transient ischemic attack, stent thrombosis, contrast volume, contrast associate acute kidney injury, BARC 3, 4 or 5 bleeding. |
| ***COMPARE 60/80 HBR*** | Patients of 18 years and above; Written or witnessed oral consent to participate in the study; Native coronary artery lesions eligible for PCI with stents with no restrictions in number of lesions and stents, vessel size or lesion complexity, apart from stent thrombosis; Patients at high risk for bleeding according to the ARC HBR criteria. | Treated with stents other than Supraflex Cruz or Ultimaster within 6 months prior to index procedure ; Treatment of lesions with stent thrombosis; Treatment of venous or arterial coronary grafts; Treated for stent thrombosis in 12 months prior to index PCI procedure; Treated with a bioresorbable scaffold 3 years before index PCI procedure; Cardiogenic shock at index procedure; Active SARS-CoV-2 infection or suspicion of SARS-CoV-2 infection; Cannot provide written informed consent; Under judicial protection, tutorship or curatorship; Unable to understand and follow study-related instructions or unable to comply with study protocol; Active bleeding requiring medical attention (BARC≥2) at index PCI; Life expectancy less than one year; Known hypersensitivity or allergy for aspirin, clopidogrel, ticagrelor, prasugrel, cobalt chromium or sirolimus; Any anticipated PCI after index PCI, unless planned and scheduled at index PCI; Participation in another stent or drug trial | (NACE) at 12-month follow-up, defined as a composite of cardiovascular death, myocardial infarction, target vessel revascularization, stroke, and major bleeding according to BARC 3 or 5 bleeding events. | Target lesion revascularization; major adverse cardiac and cerebral event, defined as the composite of cardiovascular death, myocardial infarction, and stroke; and major and clinically relevant nonmajor bleeding according to the BARC classifications: BARC 2, 3, or 5 bleeding events. |

PCI: percutaneous coronary intervention, MI: myocardial infarction, STEMI: ST elevation MI, CV: cardiovascular death, NSTEMI: non ST elevation MI; CTO: chronic total occlusion; BARC: Bleeding academic research consortium; ARC: academic research consortium; NACE: net adverse clinical events.

**Supplemental Table 3. Outcomes definitions**

| **References** | **Source for follow-up** | **CVD** | **MI** | **All-cause death** | **Stroke** | **Repeat revascularization** |
| --- | --- | --- | --- | --- | --- | --- |
| ***Cruz HBR*** | Telephone contact or an on-site visit | Death resulting from cardiovascular causes, as per the ARC-2 definition. The following categories were collected: death caused by acute myocardial infarction; death caused by sudden cardiac, including unwitnessed death; death resulting from heart failure; death caused by stroke; death caused by cardio- vascular procedures; death resulting from cardio- vascular hemorrhage; death resulting from other cardiovascular cause. | Rise of cardiac biomarker levels with at least one value above the 99th percentile upper reference limit and at least one of the following: ischemic symptoms, evidence of new ischemic changes on electrocardiogram or echocardiogram or identification of intracoronary thrombus at angiography. | All deaths were categorized as cardiovascular, non-cardiovascular, or undetermined according to ARC-2 criteria. | NS | Target lesion revascularization was defined as any repeated PCI of the target lesions including 5 mm on either side of the im- planted stent or surgical bypass of the target vessel for restenosis or other complication involving the target lesion. Target vessel revascularization was defined as any repeat PCI or surgical bypass of any segment of the target vessel. |
| ***FIRE*** | Follow-up visits occurred at one month and 12 months and were then scheduled annually for up to five years after randomization. | Cardiovascular death was defined as death resulting from cardiac causes. Among them: death for AMI, SCD, HF, stroke, cardiovascular procedures, cardiovascular hemorrhage, other cardiovascular causes. In case of undetermined cause of death, the event is classified as cardiovascular death. | The myocardial infarction is defined based on the Fourth Universal Definition of myocardial infarction. | All deaths were categorized as cardiovascular, non-cardiovascular, or undetermined according to ARC-2 criteria. | Presence of a new focal neurologic deficit thought to be vascular in origin, with signs or symptoms lasting more than 24 hours. It is strongly recommended (but not required) that an imaging procedure such as CT scan or MRI be performed. Stroke will be further classified as ischemic, hemorrhagic, or type uncertain. | Ischemia-driven coronary revascularization. Any repeated revascularization required:  • Ischemic symptoms consistent with Canadian Cardiology Society class ≥ 3 angina despite optimal medical therapy  AND  • PCI or CABG of either the culprit or a non-culprit lesion that led to enrollment into the trial plus at least one of the following: A. Positive ischemia test demonstrating clear evidence of reversible ischemia corresponding to a stenosis in a vessel other than the culprit vessel that led to enrollment into the trial, OR B. New ischemic ECG changes (ST-segment depression ≥ 1 mm, ST-segment elevation ≥ 1 mm or T wave inversion ≥ 2 mm) at rest or with exertion in a distribution consistent with a stenosis in a vessel other than the culprit vessel that led to enrollment into the trial, OR C. Fractional flow reserve (FFR) ≤0.80, instantaneous wave-free ratio (iFR)≤0.89, contrast fractional flow reserve (cFFR)≤0.85, quantitative flow ratio (QFR)≤0.80 in a lesion in a vessel other than the culprit vessel that led to enrollment into the trial. All ischemic-driven revascularizations will be sub-categorized as to whether they were due to the culprit lesion or due to a non-culprit lesion that led to enrollment into the trial. |
| ***COMPARE 60/80 HBR*** | Telephone contact or follow-up visits at 1-6-12 months after index PCI | Cardiovascular death was defined as death resulting from cardiac causes. Among them: death for AMI, SCD, HF, stroke, cardiovascular procedures, cardiovascular hemorrhage, other cardiovascular causes. In case of undetermined cause of death, the event is classified as cardiovascular death. | The myocardial infarction is defined based on the Fourth Universal Definition of myocardial infarction. | All deaths were categorized as cardiovascular, non-cardiovascular, or undetermined according to ARC-2 criteria. | Presence of a new focal neurologic deficit thought to be vascular in origin, with signs or symptoms lasting more than 24 hours. It is strongly recommended (but not required) that an imaging procedure such as CT scan or MRI be performed. Stroke will be further classified as ischemic, hemorrhagic, or type uncertain. | Target Lesion Failure is defined as cardiac death, myocardial infarction attributed to the target vessel and clinically indicated target lesion revascularization  Target Vessel Failure is defined as cardiac death, myocardial infarction attributed to the target vessel and clinically indicated target vessel revascularization  Target lesion revascularization  Urgent target vessel revascularization Non-target vessel revascularization (urgent and non-urgent) Clinically indicated target vessel revascularization |

CVD: cardiovascular death; MI: myocardial infarction; PCI: percutaneous coronary intervention. CABG: coronary artery bypass graft; FFR: fractional flow reserve; NS: not specified; AMI: acute MI; SCD: sudden cardiac death; HF: heart failure; ARC: academic research consortium; iFR: instantaneous wave free ratio; cFFR: contrast FFR; QFR: quantitative flow ratio.

**Quality appraisal**

The quality of the studies included in the meta-analysis was appraised by two unblinded reviewers (R.P. and S.B.) following the Cochrane Collaboration. For each randomized clinical trial (RCT), we evaluated the risk of selection, detection, reporting, and attrition bias (expressed as low, or high risk of bias, as well as unclear risk in case of inability to ascertain the underlying risk of bias). Results are reported in the Supplemental Figure 2. Overall, the studies included showed low risk of all biases assessed. Of note, the CruzHBR is an open label registry and not a randomized clinical trial, therefore random sequence generation may not be assessed as well as allocation concealment. Using the MINORS criteria, CruzHBR study obtained a score of (Supplemental Table 4) .

**Supplemental Figure 2. Quality assessment by Cochrane Collaboration group criteria**

**
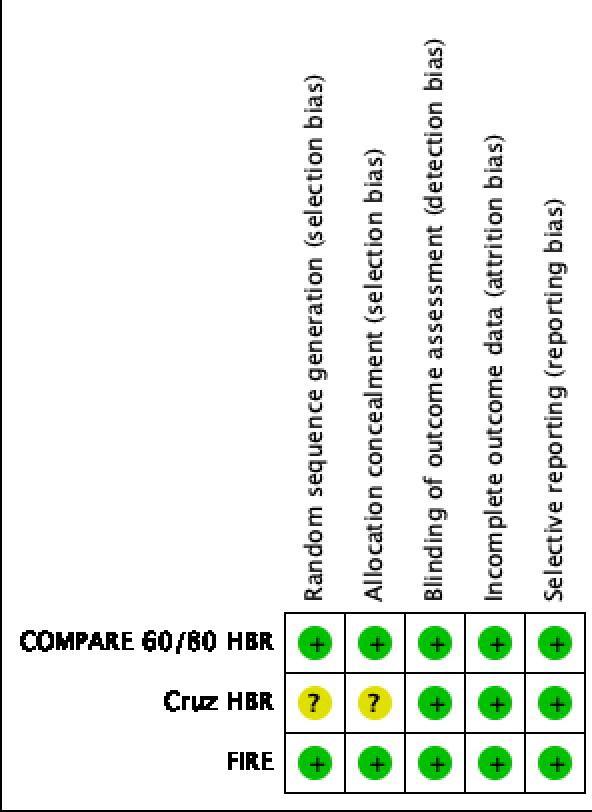
**

The Figure shows the risk of bias for each item for the studies included in the analysis.

**Supplemental Table 4. MINOR criteria for Cruz HBR**

| **References** | Q1 | Q2 | Q3 | Q4 | Q5 | Q6 | Q7 | Q8 | Q9 | Q10 | Q11 | Q12 | overall |
| --- | --- | --- | --- | --- | --- | --- | --- | --- | --- | --- | --- | --- | --- |
| Cruz HBR | 2 | 1 | 2 | 2 | 2 | 2 | 2 | 2 | 2 | 2 | 1 | 2 | 22 |

Questions (Q): 1:A clearly stated aim; 2: Inclusion of consecutive patients; 3: Prospective collection of data; 4: Endpoints appropriate to the aim of the study; 5: Unbiased assessment of the study endpoint; 6: Follow-up period appropriate to the aim of the study; 7: Loss to follow up less than 5%; 8: Prospective calculation of the study size; 9: An adequate control group; 10: Contemporary groups; 11: Baseline equivalence of groups; 12: Adequate statistical analyses. For every item: "Not reported (0 point)", "Reported but inadequate (1 point), or "Reported and adequate (2 point)" to judge. The global ideal score being 16 for non-comparative studies and 24 for comparative studies.

**Publication bias analysis**

Publication bias was appraised by graphical valuation of funnel plots, Begg and Mazumdar rank correlation, Egger’s regression intercept, and Duval and Tweedie trim and fill.

Of note, the funnel plot and all the analyses for publication bias, do not include the study of Hamza et al. because of the absence of events in the complete arm, which limited the calculation of the hazard ratio of the single study for the primary endpoint. Overall, all the tests were negative, with 0 trimmed studies (Table 5 and Supplemental Figure 3).

**Supplemental Table 5. Publication bias analyses for the primary endpoint**

|  | **Egger’s linear regression test** | | | **Begg and Mazumdar’s rank correlation test** | | **Trim and fill test** |
| --- | --- | --- | --- | --- | --- | --- |
|  | *Intercept* | *t* | *p* | *Z value for Kendall’s tau* | *p* | *N° trimmed studies* |
| *Primary endpoint* | 0.06 | 0.19 | 0.882 | 0.52 | 0.602 | 0 |

**Supplemental Figure 3. Funnel plot**


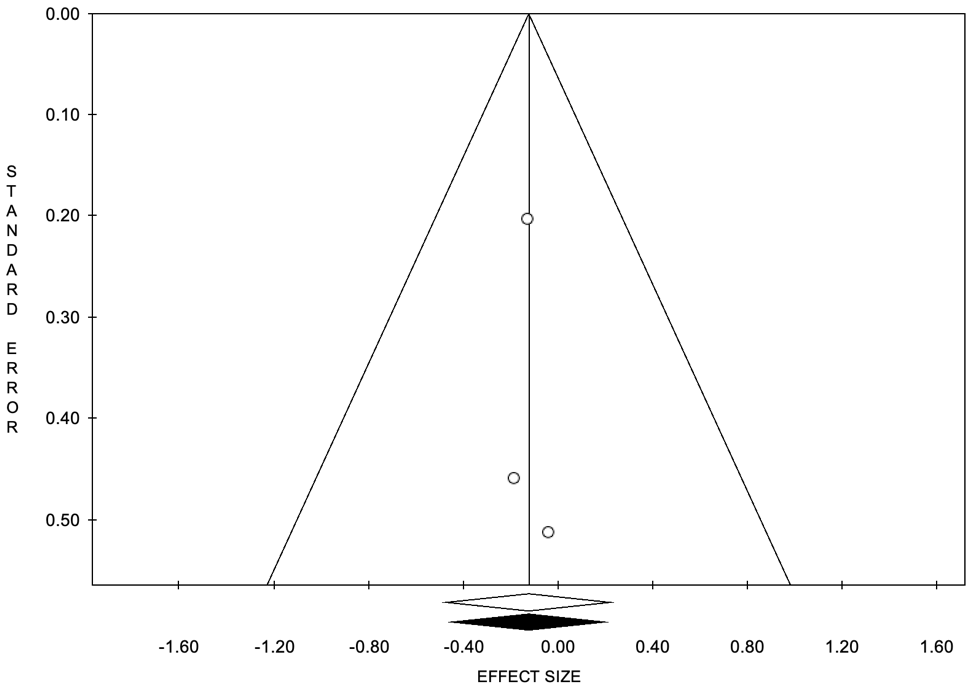


The Figure shows the analysis of the publication bias

**Supplemental Table 6. Sensitivity analysis**

|  |  | **≤30-day DAPT** | **>30-day DAPT** |  |  |
| --- | --- | --- | --- | --- | --- |
| **Outcome** | **Patients**  **no.** | **no. (%)** | **no. (%)** | **Unadjusted**  **HR (95%CI)** | **Propensity-matched**  **adjHR (95%CI)** |
| **Primary outcome** |  |  |  |  |  |
| Overall | 1692 | 89 (9.5) | 60 (7.8) | 1.21 (0.87-1.68) | 0.95 (0.67-1.33) |
| Excluding FIRE trial | 826 | 15 (4.7) | 25 (4.9) | 0.93 (0.49-1.77) | 0.91 (0.46-1.79) |
| Excluding COMPARE trial | 1326 | 79 (11.0) | 51 (8.4) | 1.30 (0.92-1.85) | 1.05 (0.73-1.53) |
| Excluding CRUZHBR trial | 1231 | 84 (10.3) | 44 (10.6) | 0.96 (0.67-1.38) | 0.84 (0.58-1.22) |
